# Ecological drivers of malaria vector habitat and transmission over 1 year of long-lasting insecticidal net intervention in Côte d’Ivoire

**DOI:** 10.1186/s13071-025-06984-9

**Published:** 2025-08-12

**Authors:** Benoit Talbot, Ludovic P. Ahoua Alou, Alphonsine A. Koffi, Colette Sih, Edouard Dangbenon, Marius G. Zoh, Soromane Camara, Serge B. Assi, Raphael N’Guessan, Louisa A. Messenger, Natacha Protopopoff, Jackie Cook, Manisha A. Kulkarni

**Affiliations:** 1 School of Epidemiology and Public Health, University of Ottawa, Ottawa, ON Canada; 2 Institut Pierre Richet (IPR)/Institut National de Santé Publique (INSP), Bouaké, Côte d’Ivoire; 3 Vector Control Product Evaluation Centre (VCPEC-IPR/INSP), Bouaké, Côte d’Ivoire; 4 Faculty of Epidemiology and Population Health, Department of Infectious Disease Epidemiology, London School of Hygiene and Tropical Medicine, London, UK; 5 Centre d’Entomologie Médicale et Vétérinaire, Université Alassane Ouattara (CEMV-UAO), Bouaké, Côte d’Ivoire; 6 Faculty of Infectious and Tropical Diseases, Disease Control Department, London School of Hygiene and Tropical Medicine, London, UK; 7 Department of Environmental and Global Health, School of Public Health, University of Nevada, Las Vegas, NV USA; 8 Department of Epidemiology and Public Health, Swiss Tropical & Public Health Institute, Allschwill, Switzerland; 9 University of Basel, Basel, Switzerland; 10 International Statistics and Epidemiology Group, London School of Hygiene and Tropical Medicine, London, UK

**Keywords:** *Anopheles*, Côte d’Ivoire, Long-lasting insecticidal nets, Machine learning, *Plasmodium falciparum*, Trial clusters

## Abstract

**Background:**

Malaria is a mosquito-borne parasitic disease that causes significant morbidity and mortality in at-risk populations, especially in children in sub-Saharan Africa. Despite reductions in malaria burden owing to the scale-up of effective interventions, there are concerns that long-lasting insecticidal net (LLIN) effects may not be sustained owing to widespread insecticide resistance and differential impacts of LLIN on vector species. In this study, we aimed to test the effect of different LLIN products and other environmental factors on the ecological niche of three mosquito vector species using state-of-the-art ecological niche modelling approaches.

**Methods:**

This study used data from a cluster randomized control trial that took place in Tiébissou, in Central Côte d’Ivoire. *Anopheles* mosquito density and *Plasmodium falciparum* vector infection data were available across 33 clusters. We used satellite remote sensing related to land cover, climate, topography and population density across the study area alongside vector species occurrence data to construct ecological niche models for *An. coluzzi*, *An. gambiae* s.s. and *An. funestus* s.s., and for *P. falciparum-*infected vectors, at baseline and 1-year post-LLIN intervention. We compared the projected habitat and habitat determinants for each species, and assessed the respective contributions of each intervention arm and environmental factors on the probability of species occurrence.

**Results:**

Minimal to considerable overall reductions in suitable habitat across the study area were observed for the three mosquito vector species (less than 1% to more than 60%), and considerable overall reduction was observed for *P. falciparum*-infected vectors (more than 50%). We did not detect an effect of intervention arm on the probability of occurrence of any vector species, while we found strong significant effects of a combination of land cover, climate, topography and/or population density variables on each of the three mosquito vector species and malaria-infected vectors. Our results suggest environmental factors may have facilitated or restricted changes in the probability of occurrence of vector species and infected vectors in the context of vector control interventions.

**Conclusions:**

Our study highlights wide ecological differences across malaria vector species and supports the need to consider malaria vector species composition when deploying malaria vector control interventions in endemic settings.

**Graphical abstract:**

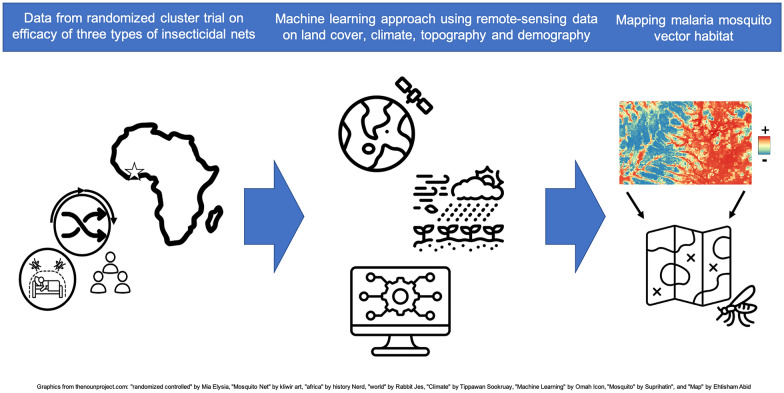

## Background

Malaria is a parasitic disease caused by eukaryotic single-cell organisms of the *Plasmodium* genus [1–3], which are transmitted by mosquitoes in the genus *Anopheles* throughout the tropical regions of the world [4–8]. *Anopheles* species responsible for human malaria transmission usually favour moderately warm [9, 10] and humid [10, 11] areas near human settlements where they can find breeding habitats and bloodmeal sources, with ecological differences across species [12, 13]. Many *Anopheles* vector species of malaria have a strong affinity for feeding upon human hosts, with variability in feeding and resting behaviours, and host preference, across species [14, 15].

*Plasmodium falciparum* is the most prevalent human malaria species in sub-Saharan Africa [16] which accounts for more than 260 million of cases and almost 600 thousand deaths each year worldwide, mostly affecting children [17–19]. The most efficient *P. falciparum* vector species belong to two mosquito species complexes, *An. gambiae* sensu lato (s.l.) and *An. funestus* s.l. Within the *An. gambiae* s.l. complex, the sibling species *An. gambiae* s.s.*, An. coluzzii* and *An. arabiensis* are important vectors [20, 21]. High ecological plasticity has been recorded in certain vector species, especially for *An. coluzzii* [22, 23], with varying levels of insecticide resistance across Africa [24–26].

As a means to control mosquito vector populations and tackle malaria burden in sub-Saharan Africa, long-lasting insecticidal nets (LLIN) have been deployed in endemic countries, contributing to the significant decline in disease transmission since 2000 [27–29]. However, different malaria vector species may respond differently to LLIN depending on the insecticide formulation, vector resistance profile, and vector ecology and behaviour, which may compromise the sustainability of malaria control in some areas [30]. Despite continuous and lengthy efforts deploying LLIN across Côte d’Ivoire [31], high levels and heterogeneity in malaria transmission continue to affect the country [32, 33]. Three of the four main African malaria mosquito vectors are present throughout the country, with *An. arabiensis* having been detected for the first time in 2022 [34], and secondary vectors *An. melas* and *An. nili* s.s. found in certain areas [35, 36]. High insecticide resistance in *An. coluzzii* and *An. gambiae* s.s. is also observed [37, 38], with new mitigation approaches continually being developed [39, 40].

Ecological niche models offer predictive information that may be useful for public health, informing on spatial distributions of habitats most suitable for disease vectors, and providing a means to predict the probability of species occurrence in different settings [41–43]. A variety of factors have been linked with habitat of the main mosquito vectors of malaria, including land cover, climate, topography and population density [44–46]. Several geostatistical and machine-learning approaches have been developed over the years, with a capacity for combining different methods into an ensemble modelling approach, greatly improving our ability to decipher patterns that would otherwise not be observable [47–49].

In this study, we aimed to test the effect of different LLIN products on the ecological niche of three mosquito vector species, *An. coluzzi*, *An. gambiae* s.s. and *An. funestus* s.s., and of *P. falciparum*-infected vectors, using state-of-the-art ecological niche modelling approaches. We used data from a cluster randomized control trial evaluating pyrethroid-piperonyl butoxide (Py-PBO) and pyrethroid-chlorfenapyr (Py-CFP) LLIN compared with a pyrethroid-only (Py-only) LLIN, from baseline and after 1 year of intervention [50]. We aimed to investigate if observable differences in the ecological niche of three vectors of *P. falciparum*, and of infected vectors, at two time points separated by 1 year, pre- and post-intervention, could be attributed to differences in intervention arms, or land cover, climate, topography and population density indices. We hypothesized that differences in vector bionomics and ecological plasticity will drive differences in the ecological niche of each vector species and how it is modified after 1 year of LLIN intervention.

## Methods

### Mosquito capture and testing data

**Fig. 1:**
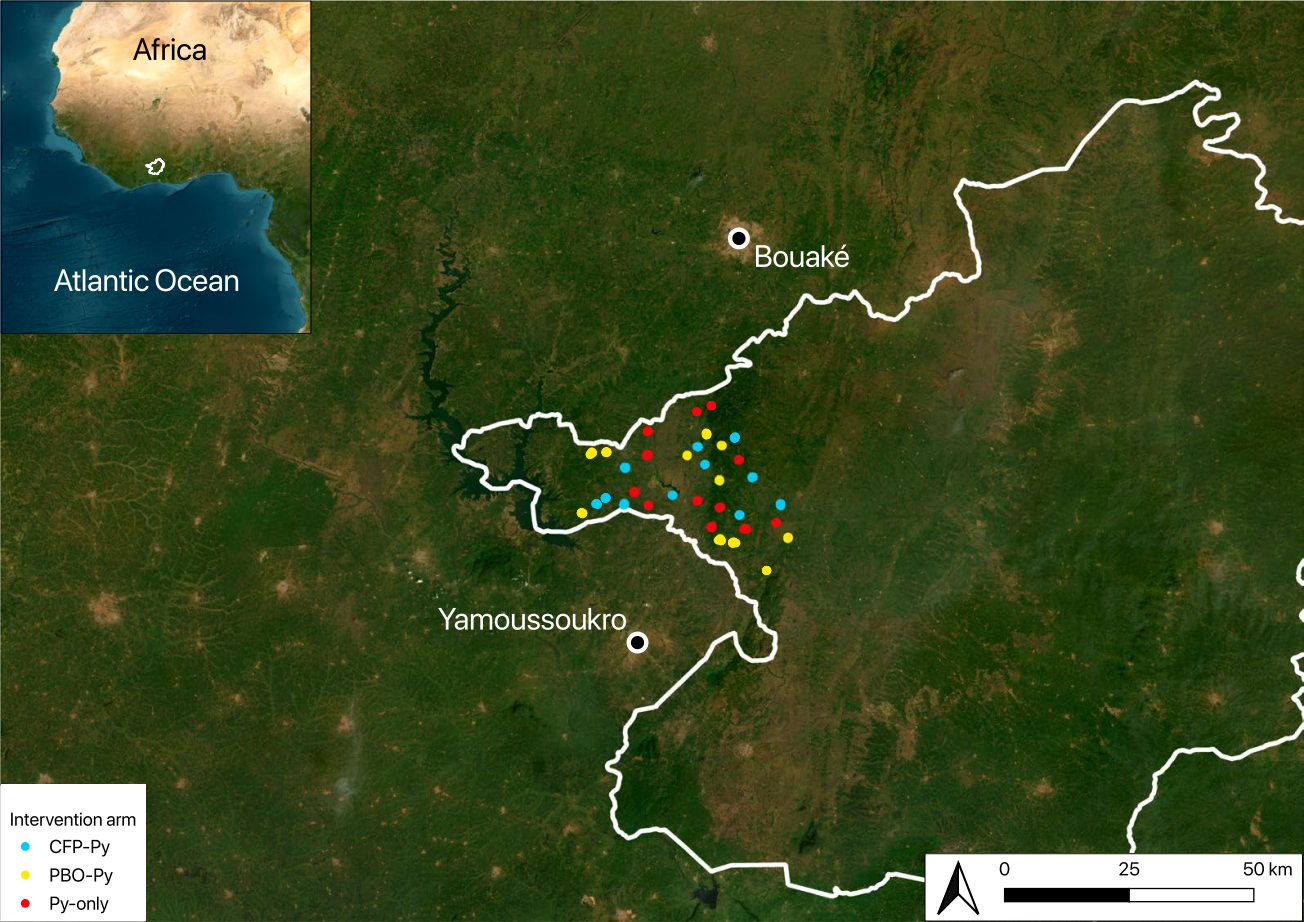
Study area showing 380 households from a cluster randomized control trial in the Tiébissou department in Côte d’Ivoire from 2023 to 2024. Inset and main maps place the Lacs district, where all study households are located, on the continent of Africa and around the study households. The capital city of Yamoussoukro and the second largest city Bouaké are identified on the main map. Intervention arm (pyrethroid-chlorfenapyr, or CFP-Py, pyrethroid-piperonyl butoxide, or PBO-Py, and pyrethroid-only, or Py-only) is identified at each household, as in legend. Map was created using QGIS 3.34.10

The study was situated in the department of Tiébissou (Lacs district), Central Côte d’Ivoire (Fig. 1). Tiébissou is about 40 km north of Yamoussoukro (political capital of Côte d’Ivoire) and approximately 60 km from Bouaké (second largest city in Côte d’Ivoire). The department of Tiébissou has 110 villages, with a population of 116,321 in 2021, spread over 2,410 km^2^. The area is a predominantly rural landscape characterized primarily by cultivated and managed vegetation (croplands) and wild vegetation (forested lands, shrublands and herbaceous lands). There is one main malaria season from May to November. The Lacs district is characterised by intense indoor malaria transmission with a prevalence of malaria reaching 51.3% in children under 5 years old, according to from the 2021 Demographics and Health Survey [51]. Mosquito net usage for the same period and age group in the Lacs district was 55.3%. Recent data in a neighbouring district showed extremely high resistance intensity to the pyrethroid insecticide deltamethrin in *An. gambiae* s.s. and *An. coluzzii* [51, 52].

A cluster randomized control trial began in July 2023 to evaluate the efficacy of two dual-active ingredient long-lasting insecticidal net (LLIN) interventions, a Py-PBO LLIN and a Py-CFP LLIN, compared with a Py-only LLIN (Clinical Trials Registration No. NCT05796193) [50]. As described by Sih et al. [50], a total of 33 clusters were allocated to one of three intervention arms using restricted randomization, resulting in 11 clusters per arm. Mosquito captures, by human landing catches, were conducted in up to six households per cluster, indoors and outdoors, starting at 18:00 and continuing until 08:00 the next morning. Collections were conducted over eight rounds approximately every 2 months between July 2023 and October 2024, with households selected randomly at each visit. Geographic coordinates were recorded in WGS84 at each household of the study. For the present work, the baseline visit, which took place from 27 July to 4 August 2023, and the post intervention visit which was conducted approximatively 1 year later, from 26 August to 10 September 2024, were used to avoid potential confounding impacts of seasonality. Therefore, we used data from a total of 380 households across the 33 clusters (Fig. 1) [50]. Characteristics of malaria vector populations from the trial’s baseline visit are reported in detail elsewhere [53].

A proportion of mosquitoes from each cluster and visit was tested molecularly to identify sibling species of *An. gambiae* s.l. and *An. funestus* s.l. and determine their infection status with the *P. falciparum* parasite. The cluster/visit-specific proportions tested were used as weights and applied to the total (indoor plus outdoor) abundance of each species for each cluster and visit to estimate the abundance of each of the three vector species and of *P. falciparum*-infected vectors.

### Land cover, climatic, topographic and demographic data

To generate environmental raster surfaces that are commonly associated with malaria vector habitat [44, 45], for use in ecological niche models, we accessed data on one class of land cover data, two classes of climatic data, two classes of topographic data and one class of demographic data at the latest year available and at the highest resolution available. We accessed data on the proportion of croplands, from the year 2019 at 100 m resolution from Copernicus Land Monitoring Service [54]. We accessed to normalized difference vegetation index (NDVI) at 375 m resolution, and land surface temperature (LST) at 1 km resolution, from US Geological Survey (USGS) [55, 56], created using Landsat 9 data. We averaged multiple 10-day NDVI measures from June to July 2023, corresponding to the wettest period in the study area, and multiple 10-day LST measures from February to March 2023, corresponding to the warmest period in the study area, using QGIS 3.34.10. We accessed elevation data from the year 2010 at 30 m resolution from Japanese Aerospace Exploration Agency (JAXA) [57], and computed slope data using QGIS 3.34.10. We accessed population density data from the year 2020 at 1 km resolution from WorldPop [58]. All data were resampled and aligned to a 100 m resolution using QGIS 3.34.10. The study area for subsequent ecological niche modelling covered an area of around 8000 km^2^ and included the entire department of Tiébissou.

### Ecological niche models

Niche modelling analysis was performed for the three main vector species (*An. coluzzii*, *An. gambiae* s.s., and *An. funestus* s.s.*)* at two time points: the baseline visit (T_0_) and the 1-year post-intervention visit (T_1_), to avoid potential confounding impacts of seasonality. We merged data for all households sampled within cluster/visit, using the geographic coordinates of the centroid of all households for each cluster/visit. All three vector species were observed at least once in the vast majority of clusters/visits. Therefore, the number of mosquitoes of each species was used to identify clusters in which species occurrence was more likely to be incidental (i.e. low habitat suitability or low probability of occurrence), and those where a resident stable population was more likely (i.e. high habitat suitability or high probability of occurrence). We calculated the threshold between presence and absence for each species as the average between the median and the first tertile of cluster-level species abundance in both T_0_ and T_1_; this approach enabled us to vary the threshold for different species to account for differences in absolute species abundance. Therefore, for *An. coluzzii*, sites with 50 or more specimens were classified as ‘presence’, and sites with 49 or less as ‘absence’, and for *An. gambiae* s.s. and *An. funestus* s.s., sites with 10 or more specimens were classified as ‘presence’, and sites with 9 or less as ‘absence’. Sites with at least 1 positive test outcome for *P. falciparum* were classified as ‘presence’, and others as ‘absence’.

The maximum entropy, random forest, and neural network algorithms were selected for our analyses, due to their demonstrated robustness for species distribution modelling [44–49]. The maximum entropy approach, using principles of parsimony and machine-learning, has been used extensively for studies predicting insect and other species distributions, including studies investigating the ecological niche of several malaria vector species throughout sub-Saharan Africa [44, 45]. The random forest decision tree-based approach, using a machine-learning alternative of a regression-based approach, has been determined to perform as well in normal conditions, and better than the maximum entropy approach in complex conditions [47, 49], and both approaches perform better than traditional regression-based approaches when using large datasets sampled over a long duration and a large spatial scale [59]. The neural network approach, using a machine-learning alternative of a probability-based approach, is a cutting-edge and powerful approach that allows better understanding of the spatial arrangements of organisms and landscapes [48]. We performed the analyses using the ‘biomod2’ package [60] in R 4.2.3 (R Development Core Team, Vienna, Austria). All spatial datasets and geographic coordinates were initially recorded in WGS84 and subsequently projected into UTM30N for analysis. We set the prevalence parameter to 0.5, meaning ‘presence’ and ‘absence’ distributions are considered in equal proportions in the analysis [61]. For each species, we trained 100 replicate models per approach, for a total of 300 replicate models, using 80% of data. To evaluate each model, we computed a receiver operating characteristic’s area under the curve (AUC) using the remaining 20% of data. Data were selected randomly in each model for training versus testing. We kept all other parameters at default values. We used all models with AUC above 0.7 to generate an ensemble niche model for each species and time point, based on committee averaging across models (i.e. the proportion of models predicting a presence) [60, 62]. We generated response plots of the mean habitat suitability index (HSI) for each explanatory variable. We calculated variable importance for each explanatory variable, which may vary from 0 to 1, using a procedure of 100 permutations from the ensemble niche model; the resulting ‘permuted importance’ value reflects the magnitude of variable contribution independent of the order in which it was entered in the model, and is therefore robust to modelling procedures. Lastly, we created a projected HSI map from the ensemble model for each mosquito species and for *P. falciparum*-infected vectors to estimate the HSI as a measure of the probability of species occurrence across the study area at 100-m resolution.

### Projected habitat

We visually compared the projected HSI maps at T_0_ and T_1_ for each species. To determine whether the projected habitat expanded, contracted or was displaced following the interventions across the study area for all studied species, we modified projected HSI maps to a binary distribution where HSI =  > 500 was considered habitat and HSI < 500 was considered non-habitat, and used the range size tool in the ‘biomod2’ package [60] in R 4.2.3.

### Habitat determinants

For each species, we subtracted the T_0_ projected HSI map from its respective T_1_ projected HSI map, resulting in a map representing HSI change from T_0_ to T_1_, using QGIS 3.34.10, with positive values reflecting a gain in species probability of occurrence between baseline and 1-year post-intervention, and negative values reflecting a loss in probability of occurrence over this period. We then sampled the values of HSI change from T_0_ to T_1_ in 500 m-radius buffers around household-specific geographic coordinates of each cluster, using QGIS 3.34.10.

To enable comparisons across species, we evaluated associations between intervention arms and HSI change for each species, calculated as the species-specific HSI change from T_0_ to T_1_ using the Py-only LLIN arm as the reference. In addition to intervention arm, we also evaluated associations between HSI change and land cover (proportion of croplands from the year 2019), climate (average hot-season LST and average wet-season NDVI in 2023), topography (elevation and slope from the year 2010) and population density (residents per km^2^ from the year 2020) variables. We standardized all variables by subtracting the mean and dividing by the standard deviation and used standard generalized linear mixed-effects models, with cluster as a random effect, to test variable effects on HSI change for all species, using R 4.2.3. We further examined model residuals to check for violations of regression assumptions and variance inflation factor to assess multicollinearity issues.

Positive regression coefficients can be interpreted as higher values of an environmental variable being associated with higher probability of species occurrence, while holding all other variables constant. Similarly, negative regression coefficients can be interpreted as higher values of environmental variables being associated with lower probability of species occurrence, while holding all other variables constant. We used a threshold *α* = 0.05 to assess variable significance and assessed model fit using the adjusted *R*^2^.

## Results

### Mosquito capture and testing data

Out of a total of 17,602 collected mosquito vector specimens, the most abundant species was *An. coluzzi* (13,168; 75% of all specimens), followed by *An. funestus* s.s. (3,207; 18%) and *An. gambiae* s.s. (1,227; 7%). On average at baseline and 1-year post-intervention across clusters, *An. coluzzii* were ten times more abundant than *An. gambiae* s.s., and four times more abundant than *An. funestus* s.s., and the same ratios were also observed for *P. falciparum*-infected mosquitoes across vector species (overall sporozoite rate ~ 2%; Table 1). There was high variation among intervention arms (Table 1).

**Table 1:** *Anopheles* mosquito collection per intervention arm and time point

Arm	Time	Total *Anopheles coluzzii*	Infected *Anopheles coluzzii*	Total *Anopheles gambiae* s.s	Infected *Anopheles gambiae* s.s	Total *Anopheles funestus* s.s	Infected *Anopheles funestus* s.s
CFP-Py	*T*_0_	115.53 (141.86)	2.71 (5.69)	8.62 (11.2)	0.14 (0.45)	24.6 (32.35)	0.64 (1.07)
	*T*_1_	133.43 (186.52)	4.44 (10.22)	4.35 (6.15)	0.67 (2.23)	4.23 (3.61)	0.1 (0.22)
PBO-Py	*T*_0_	63.7 (73.45)	0.71 (1.83)	12.48 (9.93)	0.15 (0.35)	21.96 (37.3)	0.8 (1.73)
	*T*_1_	133.62 (179.95)	1.68 (3.6)	9.16 (14.25)	0.05 (0.16)	4.46 (8.88)	0.05 (0.17)
Py-only	*T*_0_	87.65 (109.18)	0.97 (2.51)	15.8 (13.95)	0.14 (0.31)	49.82 (64.08)	1.05 (1.45)
	*T*_1_	65.59 (92.49)	1.39 (3.85)	5.46 (6.02)	0 (0)	40.73 (80.54)	0.72 (1.67)
Total		99.92 (130.57)	1.98 (4.61)	9.31 (10.25)	0.19 (0.58)	24.30 (37.79)	0.56 (1.05)

Average (standard deviation) number of individuals per cluster of *Anopheles coluzzii*, *Anopheles gambiae* s.s., and *Anopheles funestus* s.s. mosquitoes, and those infected by *Plasmodium falciparum* of each species, by intervention arm (pyrethroid-chlorfenapyr, or CFP-Py, pyrethroid-piperonyl butoxide, or PBO-Py, and pyrethroid-only, or Py-only) and time point (T_0_ and T_1_), and total across intervention arms and time points, collected during a cluster randomized control trial in the Tiébissou department in Côte d’Ivoire from 2023 to 2024, are shown

### Land cover, climatic, topographic and demographic data

**Fig. 2:**
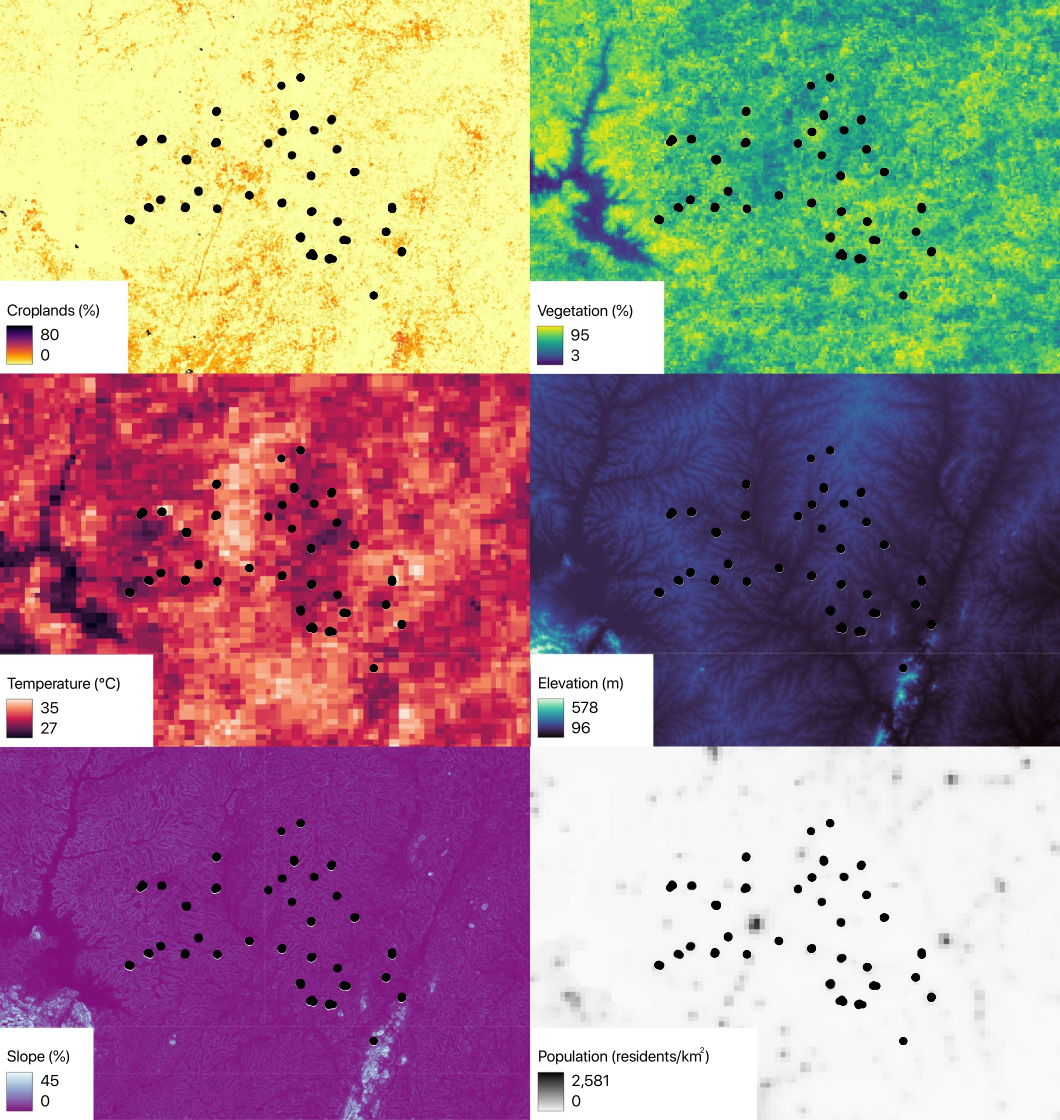
Study area showing six remote-sensing variables. Proportion of croplands (croplands), normalized difference vegetation index (vegetation), land surface temperature (temperature), elevation (elevation), slope (slope) and population density (population) across the study area are shown, as in legend. Households from a cluster randomized control trial in the Tiébissou department in Côte d'Ivoire from 2023 to 2024 are shown. Map was created using QGIS 3.34.10

The proportion of croplands, average wet-season normalized difference vegetation index (NDVI) and slope had a very wide variation across the study area (0–80%, 3–95% and 0–45%, respectively; Fig. 2). Across the study area, the hot-season land surface temperature (LST) varied from 27 to 35 °C, elevation varied from around 100–500 m, and population density varied from 0 to more than 2,500 residents per km^2^ (Fig. 2).

### Ecological niche models

**Fig. 3:**
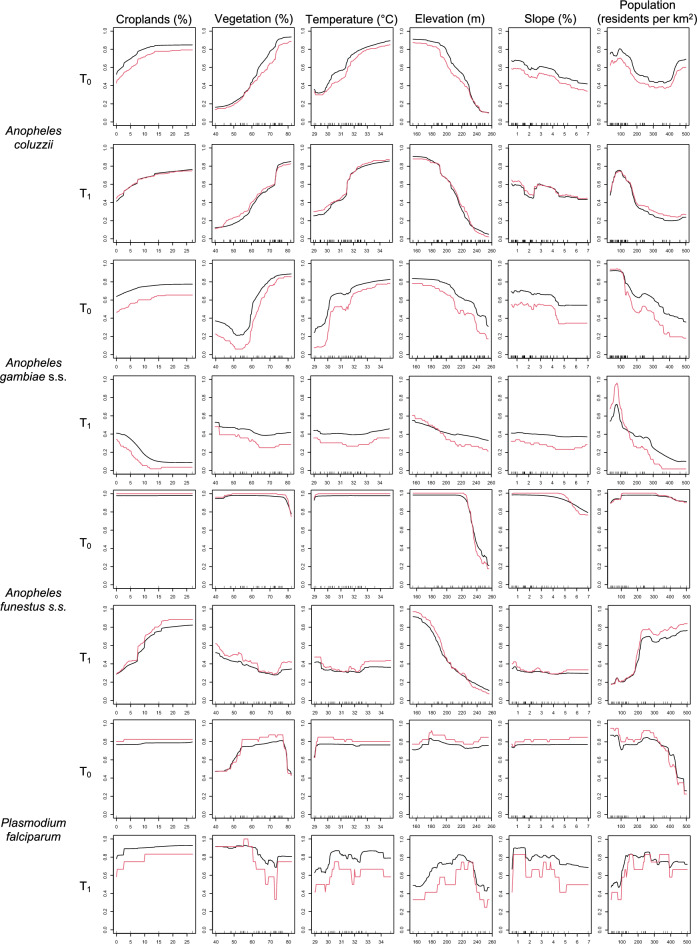
Habitat response graphs. Mean habitat suitability index (black lines) and committee averaging (red lines)of proportion of croplands (croplands), normalized difference vegetation index (vegetation), land surface temperature (temperature), elevation (elevation), slope (slope) and population density (population) at two time points (T_0_ and T_1_) for *Anopheles coluzzii*, *Anopheles gambiae* s.s. and *Anopheles funestus* s.s. mosquitoes, and *Plasmodium falciparum* isolated from the three mosquito species, collected during a cluster randomized control trial in the Tiébissou department in Côte d’Ivoire from 2023 to 2024, are shown

Area under the curve values for mean habitat suitability index (HSI) and committee averaging of the ensemble models were consistently high, approaching 1, suggesting good overall model fit (Table 2). Permuted importance of certain variables, such as LST and slope did not vary greatly between T_0_ and T_1_ and were of lesser overall importance at both time points (variable importance < 0.1). All other variables had variation in permuted importance across species and time points (Table 2), and HSI response direction also varied among species (Fig. 3).

**Table 2:** Ecological niche modelling outputs

			*Anopheles coluzzii*	*Anopheles gambiae* s.s	*Anopheles funestus* s.s	*Plasmodium falciparum*
*T*_0_	Area under the curve	Mean habitat suitability index	1.00	0.99	1.00	1.00
		Committee averaging	1.00	0.99	1.00	1.00
	Variable importance	Proportion of croplands	0.02	0.01	< 0.01	< 0.01
		Normalized difference vegetation index	0.18	0.28	0.06	0.31
		Land surface temperature	0.07	0.09	0.01	0.02
		Elevation	0.40	0.07	0.76	0.15
		Slope	0.01	0.01	0.03	0.02
		Population density	0.06	0.18	0.02	0.37
*T*_1_	Area under the curve	Mean habitat suitability index	1.00	0.98	1.00	1.00
		Committee averaging	1.00	0.95	0.99	1.00
	Variable importance	Proportion of croplands	0.01	0.15	0.13	0.03
		Normalized difference vegetation index	0.11	0.05	0.02	0.10
		Land surface temperature	0.06	0.01	0.01	0.04
		Elevation	0.45	0.15	0.41	0.15
		Slope	0.01	0.01	< 0.01	0.04
		Population density	0.09	0.61	0.39	0.16

Area under the curve of mean habitat suitability index and committee averaging values, and variable importance of six explanatory variables, for the ecological niche models performed at two time points (T_0_ and T_1_), for *Anopheles coluzzii*, *Anopheles gambiae* s.s., and *Anopheles funestus* s.s. mosquitoes, and *Plasmodium falciparum* isolated from the three mosquito species, collected during a cluster randomized control trial in the Tiébissou department in Côte d’Ivoire from 2023 to 2024, are shown

Elevation had a moderate to strong permuted importance at both time points for *An. coluzzii*, *An. funestus* s.s. and *P. falciparum*-infected mosquitoes (variable importance between 0.1 and 0.5), and a mostly negative association with HSI (Fig. 3), but a comparatively lower permuted importance for *An. gambiae* s.s. at both time points (Table 2). For *An. coluzzii*, importance of elevation increased post-intervention, and high elevations were associated with even lower HSI than pre-intervention. For *An. funestus* s.s*.*, low to mid elevation values had high HSI and high elevation values had low HSI. The variable became even more important post-intervention, and mid values became less suitable for the species. For *P. falciparum*-infected mosquitoes, elevation did not vary in importance between timepoints, but low and high values became less suitable than mid values 1 year post-intervention.

A moderate to strong permuted importance of NDVI was observed for *An. coluzzi*, *An. gambiae* s.s. and *P. falciparum*-infected mosquitoes at T_0_ (variable importance between 0.1 and 0.4), which decreased at T_1_ (Table 2). NDVI showed a mostly positive association with HSI at T_0_, except for *P. falciparum*-infected mosquitoes, and this association disappeared for all except *An. coluzzii* 1 year post-intervention (Fig. 3).

Proportion of croplands did not have a strong importance for any species at T_0_, but did have a modest permuted importance at T_1_ for both *An. gambiae* s.s. and *An. funestus* s.s., where association with HSI was opposite between the two species, i.e. negative for *An. gambiae* s.s. and positive for *An. funestus* s.s. (Table 2; Fig. 3). Similarly, population density had a modest permuted importance only for *An. gambiae* s.s. at T_0_, but its permuted importance became much more pronounced at T_1_ for *An. gambiae* s.s. and *An. funestus* s.s., where association with HSI was opposite between the two species, i.e. negative for *An. gambiae* s.s. and positive for *An. funestus* s.s. (Fig. 3). This tendency was reversed for *P. falciparum*-infected mosquitoes, where a strong permuted importance of population density at T_0_ was replaced by a modest permuted importance at T_1_, and a negative association of population density with HSI was replaced by a positive association after 1 year of intervention (Fig. 3).

### Projected habitat

**Fig. 4:**
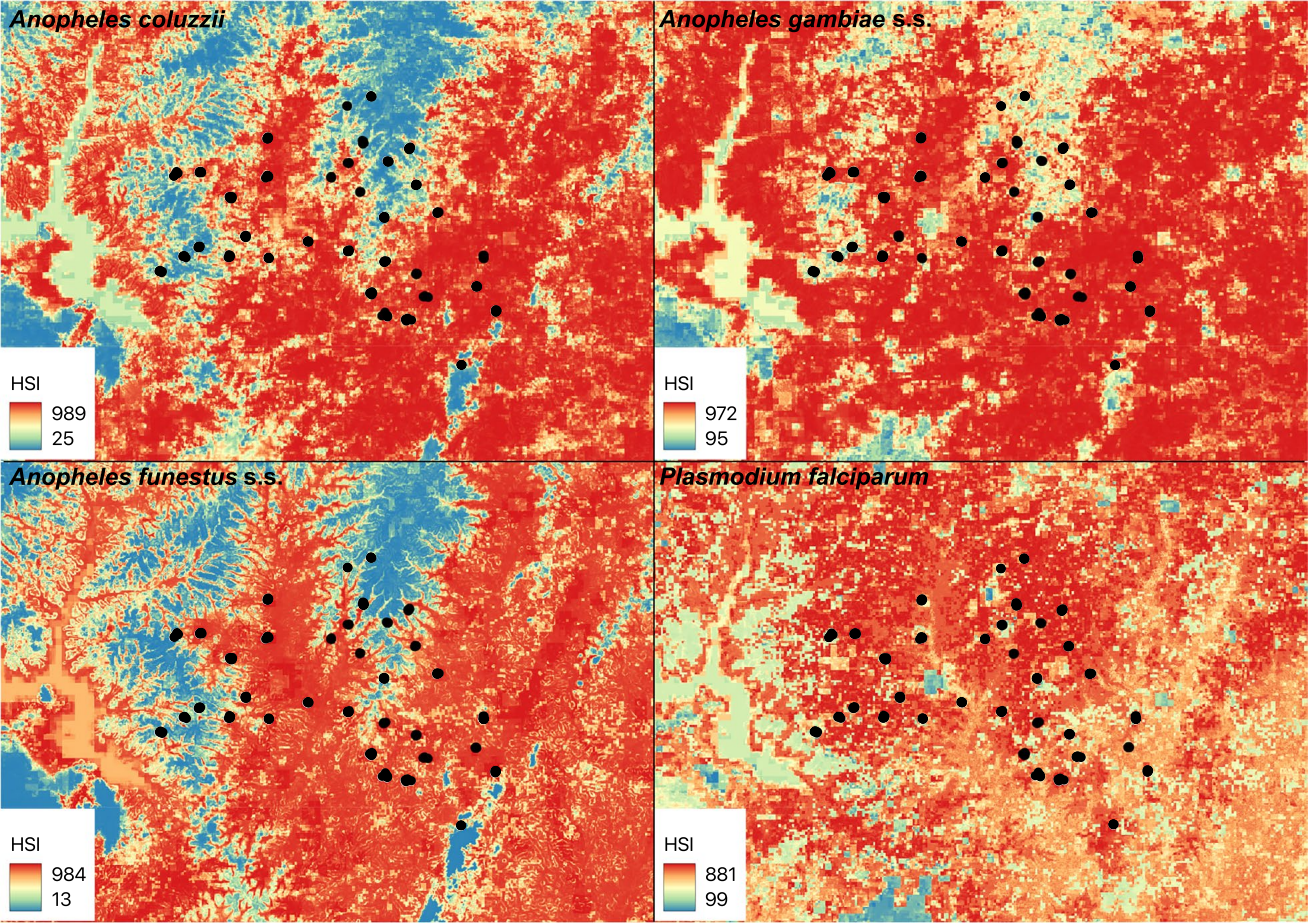
Projected habitat at the T_0_ time point. Projected habitat suitability index (HSI) at the T_0_ time point for *Anopheles coluzzii*, *Anopheles gambiae* s.s. and *Anopheles funestus* s.s. mosquitoes, and *Plasmodium falciparum* isolated from the three mosquito species, collected during a cluster randomized control trial in the Tiébissou department in Côte d’Ivoire from 2023 to 2024, is shown, as in legend. Map was created using QGIS 3.34.10

**Fig. 5:**
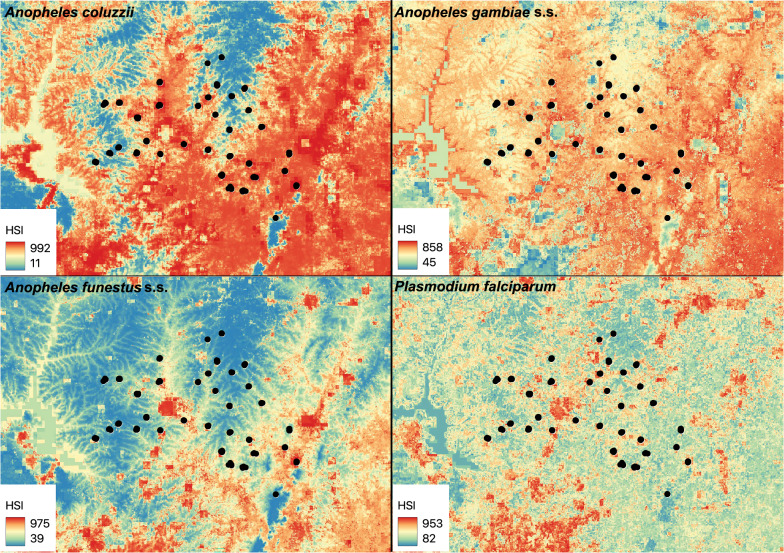
Projected habitat at the T_1_ time point. Projected habitat suitability index (HSI) at the T_1_ time point for *Anopheles coluzzii*, *Anopheles gambiae* s.s. and *Anopheles funestus* s.s. mosquitoes, and *Plasmodium falciparum* isolated from the three mosquito species, collected during a cluster randomized control trial in the Tiébissou department in Côte d’Ivoire from 2023 to 2024, is shown, as in legend. Map was created using QGIS 3.34.10

Considerable overall reduction in projected habitat from T_0_ to T_1_ across the study area was observed for *An. funestus* s.s. and *P. falciparum*-infected vectors (more than 50%; Figs. 4; 5), and moderate overall reduction in projected habitat was observed for *An. gambiae* s.s. (more than 20%). By contrast, minimal reduction in projected habitat was observed for *An. coluzzii* (less than 1%; Table 3; Figs. 4; 5).

**Table 3:** Projected habitat change from T_0_ to T_1_

Species	Loss	Gain	Range change
*Anopheles coluzzii*	4.1	3.7	−0.4
*Anopheles gambiae* s.s	26.2	3.3	−22.8
*Anopheles funestus* s.s	61.7	1.3	−60.4
*Plasmodium falciparum*	62.7	10.9	−51.7

Percent suitable habitat (habitat suitability index =  > 500) loss, gain, and change for *Anopheles coluzzii*, *Anopheles gambiae* s.s. and *Anopheles funestus* s.s. mosquitoes, and *Plasmodium falciparum* isolated from the three mosquito species, collected during a cluster randomized control trial in the Tiébissou department in Côte d’Ivoire from 2023 to 2024

### Habitat determinants

We found no significant effect of intervention arms on habitat suitability change for mosquito species and for infected mosquitoes, suggesting minimal effect of LLIN on the probability of species occurrence (Table 4).

**Table 4:** Regression modelling outputs

Species	*x*	Intervention arm		Croplands	NDVI	LST	Elevation	Slope	Population
		PBO-Py	CFP-Py						
*Anopheles coluzzii*	β	−0.649	0.333	0.022	−**0.490**	−0.026	−**0.307**	0.051	−**0.632**
	*P*	0.092	0.377	0.503	** < 0.001**	0.728	**0.005**	0.142	** < 0.001**
	*R*^2^	0.977							
*Anopheles gambiae* s.s	β	0.255	0.308	−**0.319**	−**0.469**	−**0.500**	−**0.219**	−0.021	−**0.437**
	*P*	0.401	0.313	** < 0.001**	** < 0.001**	** < 0.001**	**0.003**	0.345	** < 0.001**
	*R*^2^	0.988							
*Anopheles funestus* s.s	β	0.266	−0.346	**0.086**	**0.245**	−**0.172**	**0.447**	**0.058**	**0.380**
	*P*	0.447	0.325	** < 0.001**	** < 0.001**	**0.001**	** < 0.001**	**0.010**	** < 0.001**
	*R*^2^	0.989							
*Plasmodium falciparum*	β	0.767	0.547	**0.370**	**0.210**	**0.317**	−0.132	−0.040	**0.496**
	*P*	0.092	0.222	** < 0.001**	**0.001**	** < 0.001**	0.281	0.307	** < 0.001**
	*R*^2^	0.972							

Outputs (x: β, *P*, and *R*^2^) of effect of intervention arms (pyrethroid-chlorfenapyr, or CFP-Py, and pyrethroid-piperonyl butoxide, or PBO-Py), proportion of croplands (croplands), normalized difference vegetation index (vegetation), land surface temperature (temperature), elevation (elevation), slope (slope) and population density (population) on proportion of habitat suitability index change from T_0_ to T_1_ time points, for *Anopheles coluzzii*, *Anopheles gambiae* s.s. and *Anopheles funestus* s.s. mosquitoes, and *Plasmodium falciparum* isolated from the three mosquito species, collected during a cluster randomized control trial in the Tiébissou department in Côte d’Ivoire from 2023 to 2024, are shown. Values in bold font represent significance at *α* = 0.05

By contrast, we found strong significant effects of land cover, climate, topography and/or population density variables on HSI change from T_0_ to T_1_ on each of the three mosquito species and infected vectors (Table 4). For *An. coluzzii* and *An. gambiae* s.s., effects were all negative, suggesting that areas with higher values of environmental variables were associated with decreasing habitat suitability, or lower probability of occurrence post-intervention for these species. While we did not detect significant effects of individual LLIN intervention arms, it is possible that the effects of environmental variables amplified the combined effects of the three intervention arms on vector distributions*.* For *An. funestus* s.s. and *P. falciparum*-infected vectors, effects were mostly positive, suggesting areas with higher values of environmental variables were associated with increasing habitat suitability and probability of species occurrence post-intervention for these species (Table 4). NDVI, elevation and population density had a significant and negative effect on habitat suitability and probability of occurrence of *An. coluzzi* and *An. gambiae* s.s., while the proportion of croplands and LST also had a negative effect on *An. gambiae* s.s. (Table 4). Proportion of croplands, NDVI, elevation, slope and population density had a positive effect, and LST had a negative effect, on habitat suitability or probability of occurrence of *An. funestus* s.s. Proportion of croplands, NDVI, LST and population density had a positive effect on habitat suitability or probability of occurrence of *P. falciparum*-infected mosquitoes.

## Discussion

In this study, we aimed to investigate whether long-lasting insecticidal nets (LLIN) and other environmental factors were associated with changes in the ecological niche of three major malaria vector species, and of *P. falciparum*-infected vectors, after 1 year of intervention in a malaria-endemic region of Côte d’Ivoire.

Our study revealed differences across vector species, whereby *An. coluzzi* displayed minimal loss of projected habitat post-intervention, implying minimal change in the probability of species occurrence, while *An. gambiae* s.s. saw a moderate projected habitat loss post-intervention, and *An. funestus* s.s. saw a considerable loss in projected habitat loss post-intervention, implying moderate to considerable change in the probability of species occurrence. This supports high ecological plasticity (i.e. potential for acclimatization to new conditions) and/or rapid adaptive niche shift behaviour (i.e. potential genetic adaptation to new conditions) in *An. coluzzi* [23], which was able to maintain its broad occurrence across the study area. In contrast, the other vector species may have experienced a niche shift, a refuge effect, or a combination of the two, leading to reduced areas of occurrence [63–65]. The overall higher densities of *An. coluzzi* compared with *An. gambiae* s.s. and *An. funestus* s.s. may also partially explain the observed differences, with greater overall productivity of suitable habitats for *An. coluzzi* across the study area suggesting that this species may be better adapted to this particular setting.

Interestingly, we observed a substantial reduction in projected habitat for malaria-infected mosquitoes, of more than 50%, suggesting a general loss in area suitable for malaria transmission across the study area following LLIN intervention. This is consistent with previous malaria vector control trials in the same region and other regions in sub-Saharan Africa [66–68], although effects observed in this current study do not directly correspond to the measured trial outcomes in these other studies, such as vector density or entomological inoculation rates. While we did not detect a significant effect of LLIN interventions on probability of occurrence of vector species, the greater changes in projected habitat for *P. falciparum*-infected vectors compared with vectors themselves is of interest. This may relate to potential effects of LLINs on parasite development within mosquitoes, as has been observed for dual active ingredient LLIN that incorporate chlorfenapyr along with a pyrethroid [69], or other LLIN effects on vector survival that impact the sporozoite rate [66]. While it may also be attributed to patterns in insecticide resistance intensity in vector populations [24–26], populations of *An. coluzzii* and *An. funestus* s.s in this area were characterized by intense pyrethroid resistance at trial baseline and 1-year post-intervention, and there was no notable escalation in resistance intensity during this period.

Certain environmental variables seem to have accentuated or restricted the observed loss of projected habitat for malaria vectors species and infected vectors, while the direction and magnitude of their effects varied across species. Our results suggest *An. coluzzii* experienced a shift out of areas with high NDVI and high elevation into areas with low and moderate levels of NDVI and elevation. Our results also suggest *An. gambiae* s.s. experienced a shift out, or was removed, from areas with moderate to high proportion of croplands, NDVI and population density, and either shifted into or persisted in areas with low values of all these variables. In rural habitats, *An. coluzzii* typically exhibits ecological plasticity in its response to elevation, which is not as prominent in *An. gambiae* s.s. [22]. Moreover, *An. gambiae* s.s. (historically the S form of *An. gambiae* s.l.) typically thrives in rain-fed puddles, whereas *An. coluzzii*, (historically named the M form of *An. gambiae* s.l.), thrives in human-made ponds, and therefore is less dependent on rainfall [70]. Higher rainfall, for which NDVI may be used as a proxy, is associated with higher activity of *An. gambiae* s.l., comprising both *An. coluzzii* and *An. gambiae* s.s. [71], but excessive rainfall may cause larval loss [72]. Our findings suggest *An. coluzzii* and *An. gambiae* s.s. were displaced from areas that are generally at the limit of their ecological tolerance. For *An. funestus* s.s., our findings suggest low-elevation areas were suitable overall, high-elevation areas were unsuitable overall, and moderate elevation became less suitable after intervention. Our findings also suggest areas with low values of proportion of croplands and population density became unsuitable after intervention. Typical habitat of *An. funestus* s.s. is low-lying riverine habitats in proximity to human villages, where they can access permanent or semi-permanent breeding sites [71], and the species shows high population genetic structure possibly owing to low long-distance migration behaviour or capacity [74]. These characteristics of *An. funestus* s.s. would support a lesser propensity to evade the LLIN interventions, and persistence of the species in areas that are generally more suitable for it [63]. For *P. falciparum*-infected mosquitoes, highly vegetated and highly populated areas may have constituted refuges. Our findings suggest that proximity to humans, who are the reservoir of *P. falciparum*, and shelter for vector breeding and resting habitats provided by vegetation, may have led to persistence of the parasite in localized vector populations [75].

Our study is subject to some limitations. To detect the effects of different LLIN types on vector species probability of occurrence, we used the pyrethroid-only LLIN arm as a reference in our analysis; however, it is possible that Py-only nets had some impact in their first year, particularly as net coverage prior to the trial was lower (approximately 38% household LLIN access in June 2023) [76]. The analysis also suffers from low statistical power due to low number of trials clusters, which is partially mitigated by state-of-the-art methods that can utilize the observed variation to its full extent. Future research would benefit from larger datasets, assessments over areas with greater environmental variability and/or evaluations over longer time periods.

## Conclusions

Our results support an overall reduction of projected habitat and probability of occurrence for two of the three mosquito vector species, *An. gambiae* s.s. and *An. funestus* s.s., and change was more pronounced for mosquitoes infected by *P. falciparum*. Over the 1-year intervention period, we saw a decrease in suitable areas for malaria transmission associated mainly with proportion of croplands, NDVI, elevation and population density. Our findings highlight wide ecological differences and response to intervention across vector species, and supports a species-specific approach to intervention.

## Data Availability

Data supporting the main conclusions of this study are included in the manuscript.
