# Correction: Ecological drivers of malaria vector habitat and transmission over 1 year of long-lasting insecticidal net intervention in Côte d’Ivoire

**DOI:** 10.1186/s13071-025-07023-3

**Published:** 2025-09-10

**Authors:** Benoit Talbot, Ludovic P. Ahoua Alou, Alphonsine A. Koffi, Colette Sih, Edouard Dangbenon, Marius G. Zoh, Soromane Camara, Serge B. Assi, Raphael N’Guessan, Louisa A. Messenger, Natacha Protopopoff, Jackie Cook, Manisha A. Kulkarni

**Affiliations:** 1 School of Epidemiology and Public Health, University of Ottawa, Ottawa, ON Canada; 2 Institut Pierre Richet (IPR)/Institut National de Santé Publique (INSP), Bouaké, Côte d’Ivoire; 3 Vector Control Product Evaluation Centre (VCPEC-IPR/INSP), Bouaké, Côte d’Ivoire; 4 Faculty of Epidemiology and Population Health, Department of Infectious Disease Epidemiology, London School of Hygiene and Tropical Medicine, London, UK; 5 Centre d’Entomologie Médicale et Vétérinaire, Université Alassane Ouattara (CEMV-UAO), Bouaké, Côte d’Ivoire; 6 Faculty of Infectious and Tropical Diseases, Disease Control Department, London School of Hygiene and Tropical Medicine, London, UK; 7 Department of Environmental and Global Health, School of Public Health, University of Nevada, Las Vegas, NV USA; 8 Department of Epidemiology and Public Health, Swiss Tropical & Public Health Institute, Allschwill, Switzerland; 9 University of Basel, Basel, Switzerland; 10 International Statistics and Epidemiology Group, London School of Hygiene and Tropical Medicine, London, UK

**Correction: Parasites & Vectors (2025) 18:343** 10.1186/s13071-025-06984-9

Following publication of the original article [1], it came to the publisher’s attention that the images of Fig. 3 were misaligned because of a typesetting error during production of the article. The figure has now been corrected. Thank you for reading this erratum and apologies for any inconvenience caused.
